# High strength and low swelling composite hydrogels from gelatin and delignified wood

**DOI:** 10.1038/s41598-020-74860-w

**Published:** 2020-10-20

**Authors:** Shennan Wang, Kai Li, Qi Zhou

**Affiliations:** 1 Division of Glycoscience, Department of Chemistry, School of Engineering Sciences in Chemistry, Biotechnology and Health, KTH Royal Institute of Technology, AlbaNova University Centre, 106 91 Stockholm, Sweden; 2 Wallenberg Wood Science Center, Department of Fibre and Polymer Technology, KTH Royal Institute of Technology, 100 44 Stockholm, Sweden

**Keywords:** Composites, Mechanical properties, Gels and hydrogels

## Abstract

A delignified wood template with hydrophilic characteristics and high porosity was obtained by removal of lignin. Gelatin was infiltrated into the delignified wood and further crosslinked with a natural crosslinker genipin to form hydrogels. The composite hydrogels showed high mechanical strength under compression and low swelling in physiological condition. The effect of genipin concentrations (1, 50 and 100 mM) on structure and properties of the composite hydrogels were studied. A porous honeycomb structure with tunable pore size and porosity was observed in the freeze-dried composite hydrogels. High elastic modulus of 11.82 ± 1.51 MPa and high compressive yield stress of 689.3 ± 34.9 kPa were achieved for the composite hydrogel with a water content as high as 81%. The equilibrium water uptake of the freeze-dried hydrogel in phosphate buffered saline at 37 °C was as low as 407.5%. These enables the delignified wood structure an excellent template in composite hydrogel preparation by using infiltration and in-situ synthesis, particularly when high mechanical strength and stiffness are desired.

## Introduction

Recently, utilizing the inherent nanocellulose fibril orientation, anisotropic tissue organization and cellular structure in wood, synthetic polymers and inorganic materials have been introduced into delignified wood to generate composites with transparency, electrical conductivity, and novel functions for applications in load-bearing materials, environmental remediation, actuators, solar steam generation, etc.^1–4^. Wood-based hydrogels have also been synthesized by filling the micro channels of delignified wood with the polyacrylamide (PAM) hydrogel precursor followed by free-radical polymerization^5^. The wood/PAM hydrogel demonstrated remarkably enhanced fracture tensile strength and modulus as compared to the PAM hydrogel owing to the skeletons of natural aligned cellulose nanofibers (CNFs) and the strong interfacial hydrogen bonding in the hydrogel. This top-down approach opened up a new way to synthesize strong hydrogels exploiting the advantages of the cellular structure with highly aligned CNFs bundles in the cell walls in delignified wood, which provides not only mechanical support but also liquid conduction through the natural aligned micro and nano channels.

Gelatin is a mixture of peptide hydrolysed from collagen and has been widely used in food, pharmaceutical and cosmetic industries as a stabilizing ingredient since it is non-toxic, biocompatible, and biodegradable. In tissue engineering and biomedicine, gelatin-based hydrogels with porous network structures can provide an ideal environment for cells attachment and promote their colonization, migration and proliferation^6–8^. The gelation of mammalian and warm-water fish gelatin in aqueous solution occurs at temperature below 25 °C due to physical folding of the polypeptide backbone, i.e. the single-strand to triple-helix transition of gelatin chains. The gelling temperature of cold-water fish gelatin is even lower, at around 10 °C^9,10^. This gelation process is reversible as the temperature increases to 37 °C. To avoid the dissolution of gelatin at the physiological temperatures, chemical or enzymatic crosslinking is usually employed^11–13^. Conventional synthetic crosslinking reagents such as glutaraldehyde, diisocyanates, and carbodiimides, as well as genipin, a natural crosslinker with low acute toxicity, have been used for gelatin crosslinking. However, the poor mechanical performance and high swelling ratio of gelatin largely limit the application of gelatin hydrogels.

The strategies to substantially improve mechanical properties of hydrogel include hybridization with nanomaterials or incorporation of multiple components with different crosslinking mechanisms involving chemical bonding and physical entanglement^14^. Various matrixes, either organic or inorganic, have been hybridized with gelatin to improve its mechanical and thermal performance^15–19^. For example, crosslinked porous gelatin/chitosan scaffolds with controllable mechanical properties for load bearing soft tissues have been prepared using genipin^20^. Formation of interpenetrating network (IPN) and semi-IPN structures in gelatin/chitosan hydrogels could also enhance the mechanical properties. Gelatin was modified with methacrylic anhydride and covalently crosslinked by ultraviolet light radiation, while chitosan was crosslinked by hydrophobic interactions at high pH^19^. Gelatin/alginate hydrogels with IPN structures by chemical crosslinking of gelatin with genipin or carbodiimide together with ionic crosslinking of alginate with calcium or zinc ions have also been prepared. The hydrogel was further reinforced with cellulose nanocrystals (CNCs) and demonstrated good mechanical properties comparable to natural cartilage^21,22^. Dialdehyde CNCs prepared by sodium periodate oxidation was able to crosslink gelatin to form hydrogel with improved mechanical properties and thermal stability^23^. CNFs have also been dispersed into crosslinked gelatin/chitosan matrix to fabricate 3D porous scaffolds by freeze-drying^24^. The scaffolds demonstrated high porosity and viscoelasticity when immersed in phosphate buffered saline (PBS). Bacterial cellulose (BC)/gelatin composite hydrogels crosslinked with *N*-(3-dimethy-laminopropyl)-*N*′-ethylcarbodiimide hydrochloride have also showed significant enhancement in mechanical strength and stiffness owing to the natural ultrafine-fibre network structures of BC^25–27^. The fracture stress and elastic modulus of BC/gelatin hydrogel under compression were reported to be 3.7 MPa and 1.7 MPa with water content of 82.8 wt%, respectively^27^. The interfacial interaction and bonding between cellulose and gelatin and the formation of CNF network structure are important to the mechanical properties of gelatin/CNFs hydrogels. To further increase their mechanical properties while maintaining high water content (> 80 wt%), the formation of oriented structure of aligned CNFs in the hydrogel is highly desirable. However, this is rather challenging and requires shear-induced alignment when using CNFs by bottom-up assembly approaches^28,29^.

**Figure 1:**
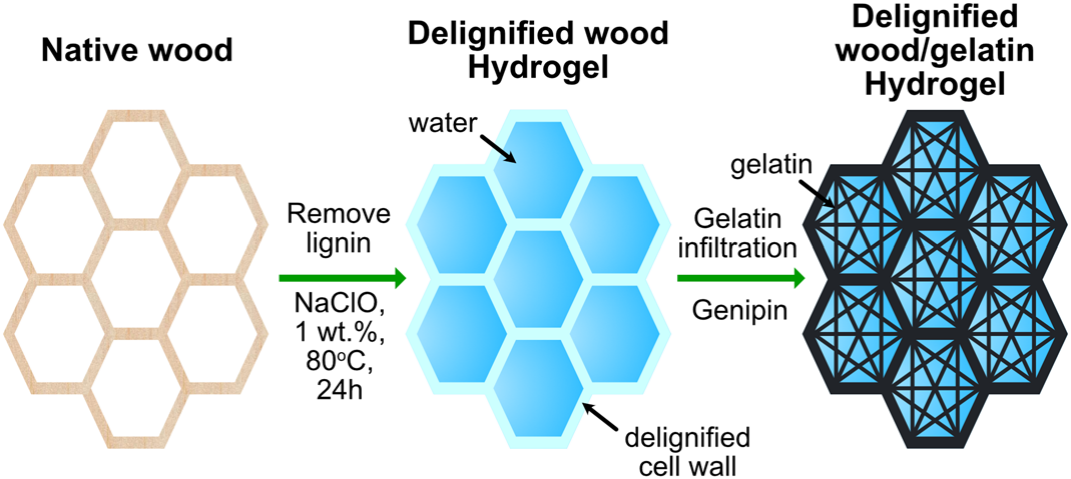
Schematic illustration for the preparation of delignified wood/gelatin composite hydrogel.

In this work, delignified wood with inherently aligned CNFs and native porous cell wall structure is used as mechanical reinforcement and structural confinement for gelatin hydrogels, aiming to enhance the compressive mechanical properties of gelatin hydrogel as well as decreasing its swelling under physiological condition. Composite hydrogels from delignified wood and gelatin have been prepared by infiltration of gelatin followed by chemical crosslinking with genipin at different concentrations and characterized in terms of structural features (porosity and pore size), molecular configuration, mechanical properties and swelling behaviour. As illustrated in Fig. 1, lignin was removed from wood structure and the cellular structure with inherent CNFs alignment in the cell wall was preserved. Gelatin was infiltrated in the channels of the delignified wood and crosslinked by genipin. The microstructure of the delignified wood/gelatin composite hydrogels was characterized by optical microscopy and field emission scanning electron microscopy (FE-SEM). The compressive mechanical properties and the swelling behaviour of the composite hydrogels were studied.

## Results and discussion

### Structure of the composite hydrogel

**Figure 2:**
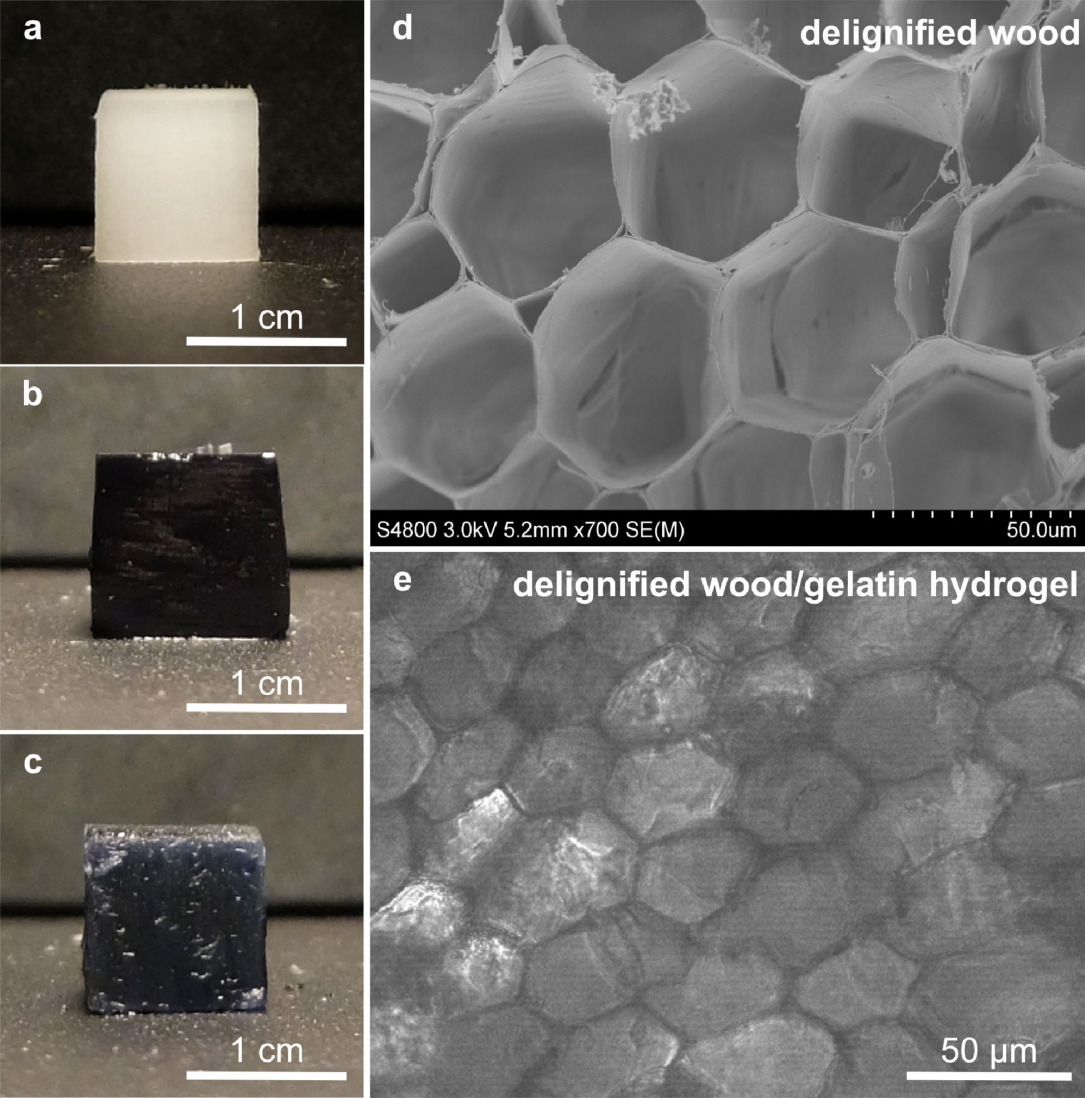
Photographs of (**a**) delignified wood, (**b**) gelatin and (**c**) delignified wood/gelatin hydrogel. (**d**) SEM image of freeze-dried delignified wood, showing the well-preserved cellular structure. (**e**) Optical microscopy image of delignified wood/gelatin composite hydrogel in wet state with initial gelatin and genipin concentrations of 30 wt% and 100 mM.

**Figure 3:**
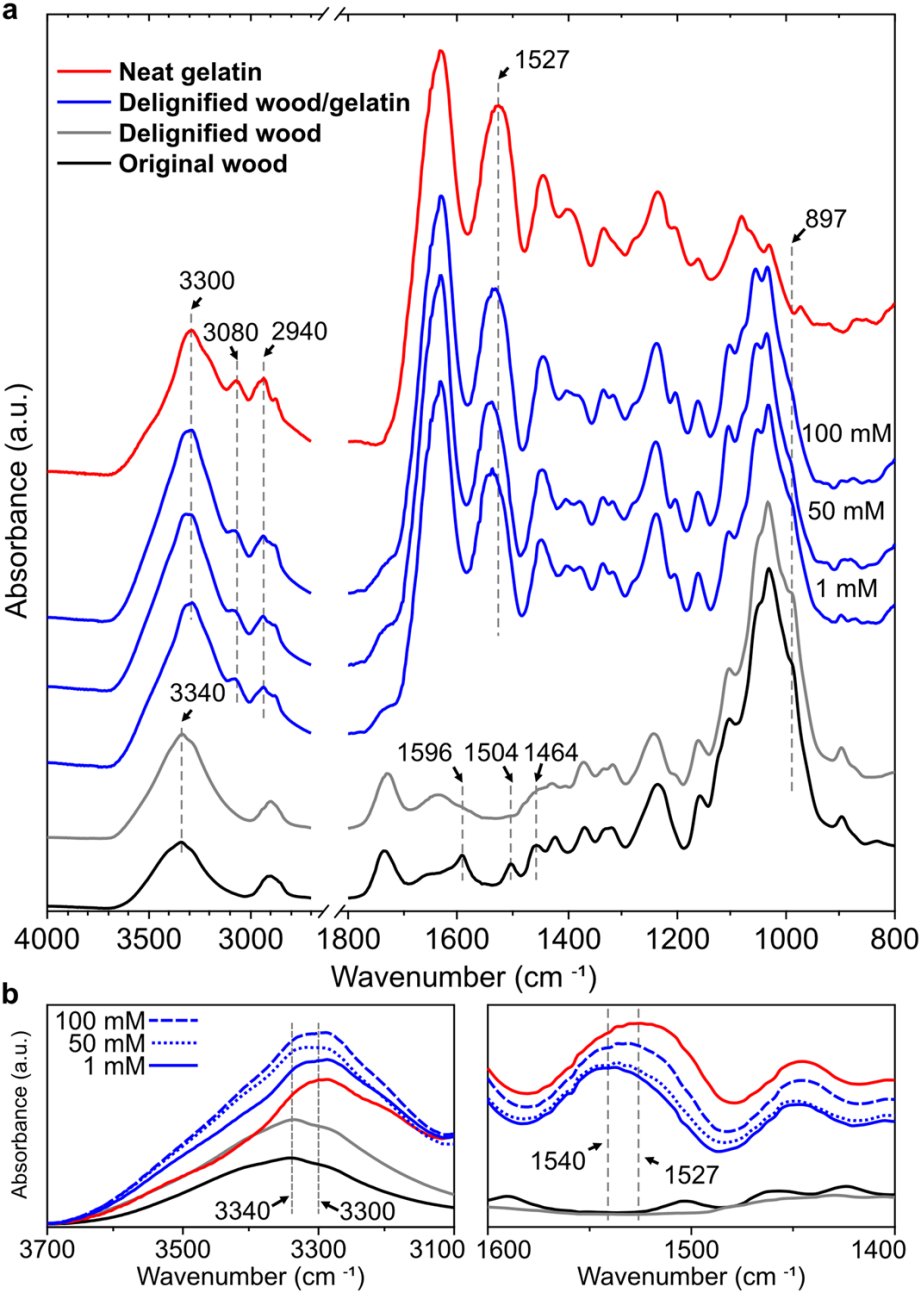
ATR-FTIR spectra of original wood, delignified wood, neat gelatin and delignified wood/gelatin hydrogel crosslinked with genipin at different concentrations in the range of (**a**) 4000 to 800 cm^-1^ and (**b**) 3700 to 3100 cm^-1^ and 1600 to 1400 cm^-1^.

Balsa wood was delignified by using 1 wt% sodium chlorite at 80 °C. The main functional substance in sodium chlorite delignification is chlorine dioxide, which has been widely used for disinfection, sterilization in food and medical application owing to its low toxicity. The delignified wood was washed intensively with deionized water before the infiltration of gelatin. Delignified wood showed a white colour (Fig. 2a) as most of lignin and chromophores have been removed from the wood structure. This was further confirmed by the disappearing of lignin-attributed peaks^30^ at 1596 cm^−1^ (symmetric ring stretching), 1504 cm^−1^ (asymmetric ring stretching) and 1464 cm^−1^ (asymmetric C–H deformation) in the ATR-FTIR spectrum of delignified wood as compared to the original Balsa wood (Fig. 3). Besides, hemicelluloses remained in the delignified wood as indicated by the peak at 1730 cm^−1^ corresponding to the C=O stretching frequency of carbonyl groups in hemicellulose.

The crosslinking strategies significantly impact the structure and properties of gelatin-based hydrogel^31,32^. The complete gelation of gelatin (37.5 wt%) was achieved within 35 min at 37 °C when 1 wt% (44 mM) genipin was used as a crosslinker^33^. However, the complete infiltration of gelatin into porous delignified wood structure takes much longer time. To avoid gelling, gelatin from bovine skin was first infiltrated into the delignified wood at 50 °C for one week. The composites were then kept overnight at 4 °C, which is below the upper critical solution temperature of gelatin, to allow physical gelation of gelatin. Subsequently, the composite hydrogels were further chemically crosslinked in a genipin solution for 48 h, to assure complete crosslinking of infiltrated gelatin in the composite. The initial genipin concentration and the compositions of gelatin, delignified wood, and water in the resulting composite hydrogels are summarized in Table 1. The delignified wood/gelatin composite hydrogels with water content of around 81 wt% were successfully prepared. The water content was lower than those for neat gelatin hydrogel due to the interaction between gelatin and delignified wood and the structural confinement provided by the wood cell wall structure as evidenced by FTIR analysis and swelling behaviour discussed in latter sections. Nonetheless, a highly hydrated network (water content > 80 wt%) was maintained in the composite hydrogels, which is of great interests for ultra-stiff/strong hydrogel preparation^34^. Both gelatin hydrogel and the delignified wood/gelatin hydrogels showed a dark blue colour (Fig. 2b,c) indicating the successful chemical crosslinking of gelatin by genipin. This characteristic colour is typically observed for dyeing in food, textile, and tattoo when the amine groups are crosslinked with genipin. Infiltration of gelatin into the lumen of the wood structure was confirmed by the microscopy observation (Fig. 2d,e). This was further confirmed by FTIR analysis (Fig. 3). The absorption band at 1527 cm^-1^ in neat gelatin was ascribed to NH bending (amide II). The shift of the amide II band from 1527 to 1540 cm^-1^ (Fig. 3b) was observed in delignified wood/gelatin hydrogels, indicating that the presence of delignified wood prevented the formation of β-sheet structure, resulting in more random coil to α-helix structure during the physical folding of gelatin in cooling process^35^. The peak at 897 cm^-1^ (Fig. 3a) is originated from the CH deformation at C1 position associated with C–O–C stretching of the β-glycosidic linkages in cellulose and hemicellulose^36^. The intensity of the shoulder peak at 897 cm^-1^ in the composite hydrogels decreased with increasing crosslinking density at higher genipin concentrations, which indicating an increased hydrogen bonding between gelatin and delignified wood. Indeed, the broadening of the OH stretching vibration region centred at 3340 cm^-1^ (Fig. 3b) and the NH stretching of hydrogen-bonded amide group at 3300 cm^-1^ was observed for the composite hydrogel with increasing genipin concentration^37^. The peaks at 3080 and 2940 cm^-1^ (Fig. 3a) were attributed to the CH_3_ stretching and CH_2_ stretching of amide B structure, respectively^38^. The relative intensity between 3080 to 2940 cm^-1^ (*I*_*3080*_/*I*_*2940*_) of genipin crosslinked delignified wood/gelatin composite was higher than the neat gelatin, indicating stronger entanglement in the gelatin network.

**Table 1:** Initial genipin concentration and the composition of gelatin, delignified wood, and water in the resulting hydrogels.

Sample	Genipin (mM)	Gelatin (wt.%)	Delignified wood (wt.%)	Water (wt.%)
Gelatin hydrogels	1	11.8 ± 0.1	–	88.2 ± 0.1
	50	13.4 ± 0.2	–	86.6 ± 0.2
	100	14.1 ± 0.1	–	85.9 ± 0.1
Delignified wood/Gelatin hydrogels	1	14.0 ± 0.1	4.8 ± 0.1	81.2 ± 0.8
	50	14.2 ± 0.1	4.9 ± 0.1	80.9 ± 0.3
	100	14.4 ± 0.1	4.8 ± 0.1	80.8 ± 0.6
Delignified wood	–	–	5.5 ± 0.4	94.5 ± 0.4

**Figure 4:**
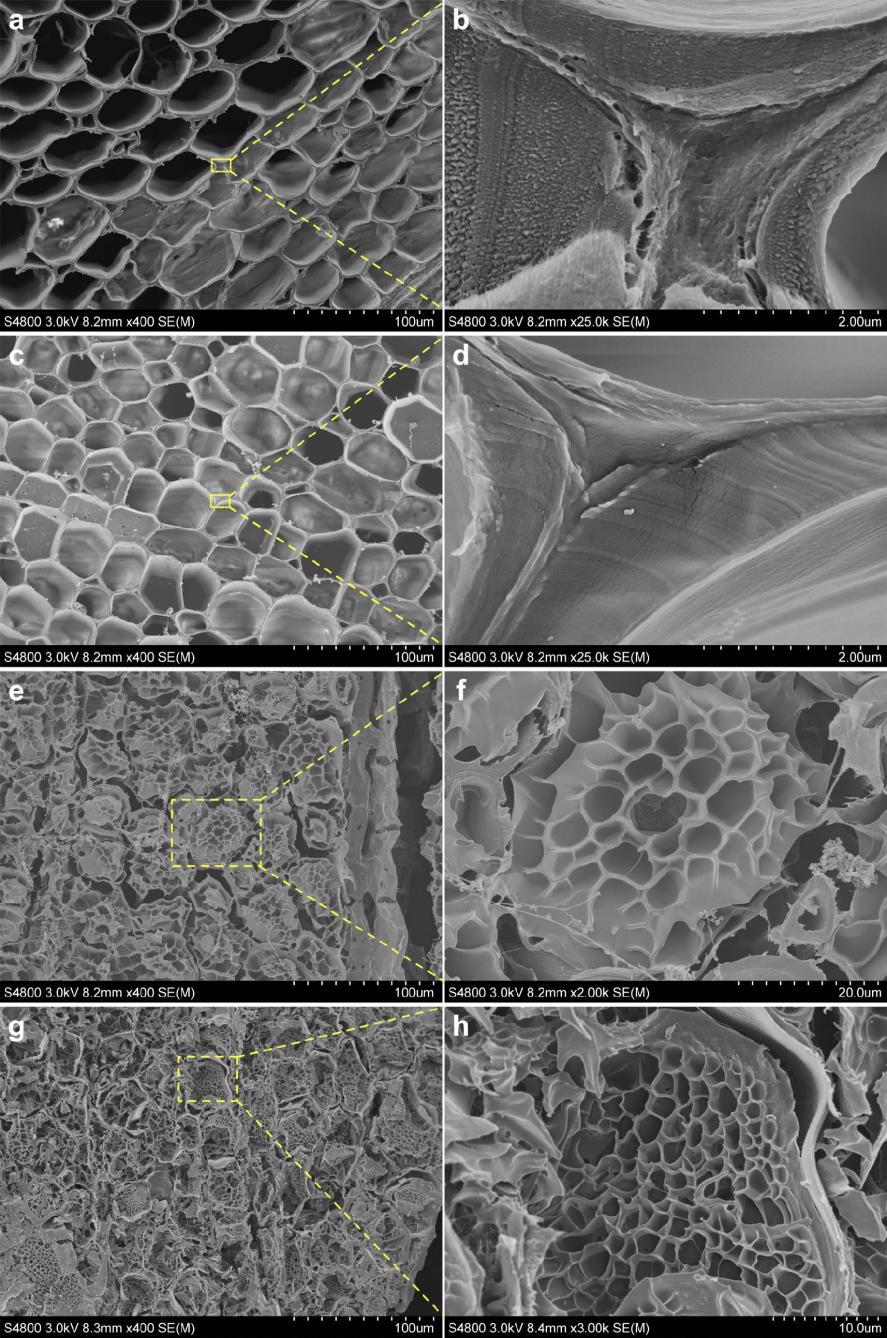
Cross section SEM images of freeze-dried delignified wood (**a**), (**b**) and freeze-dried delignified wood/gelatin hydrogel crosslinked with different genipin concentrations: (**c**), (**d**) 1 mM; (**e**), (**f**) 50 mM; (**g**), (**h**) 100 mM.

The effect of genipin concentrations on the morphology and pore structure of delignified wood/gelatin hydrogels was further studied by FE-SEM (Fig. 4) using freeze-dried samples. Their pore size and porosity data are summarized in Table 2. As shown in Fig. 4a, the cross section of delignified balsa wood possessed a honeycomb-like structure which is composed of hollow fiber cells with diameters between 20 and 50 μm. Nano- and micron-scale pores were observed in both cell walls and cell wall corners (Fig. 4b), and a total porosity of 93.4 ± 0.6% was obtained. Such porous structure allowed the penetration of gelatin into the cell wall structure and facilitated the interaction of gelatin with cellulose and hemicellulose in delignified wood. The fixation of gelatin in delignified wood was enabled by chemical crosslinking with genipin. When only 1 mM genipin solution was applied, the composite hydrogel shows similar macroscopic structure as delignified wood (Fig. 4c). Due to the low initial concentration of genipin, network structure of gelatin did not form in the lumens. Gelatin was mainly adsorbed onto the surface of lumens and infiltrated into the cell wall and cell wall corners, resulting in a decreased porosity of 83.7 ± 0.2%. Indeed, smooth surface was observed at both cell wall and cell wall corners (Fig. 4d). When the genipin concentration was increased to 50 mM, gelatin network was formed within lumens and showed a pore size of 6.0 ± 1.2 μm (Fig. 4e,f). As the concentration of genipin was increased to 100 mM, denser gelatin network was clearly observed in lumens (Fig. 4g,h), and the crosslinked gelatin aerogel exhibits a porous structure with relatively smaller pore size of 2.3 ± 0.5 μm. Consequently, the total porosity of delignified wood/gelatin hydrogel decreased to 72.7 ± 0.3% and 69.0 ± 0.2% when 50 and 100 mM genipin were applied, respectively. Beyond short-range crosslinking between amino acids, genipin also undergoes a long-range intermolecular crosslinking which involves self-crosslinking between genipin monomers. By tuning the degree of polymerization of polygenipin, the pore size of gelatin scaffold certainly can be controlled for different purposes^39^.

**Table 2:** Porosity of freeze-dried delignified wood/gelatin hydrogels and pore size of the infiltrated gelatin inside the lumens of delignified wood.

Sample	Genipin (mM)	Pore size (μm)	Porosity (%)
Delignified wood/ Gelatin hydrogels	1	–	83.7 ± 0.2
	50	6.0 ± 1.2	72.7 ± 0.3
	100	2.3 ± 0.5	69.0 ± 0.2
Delignified wood	–	–	93.4 ± 0.6

### Compressive mechanical properties

**Figure 5:**
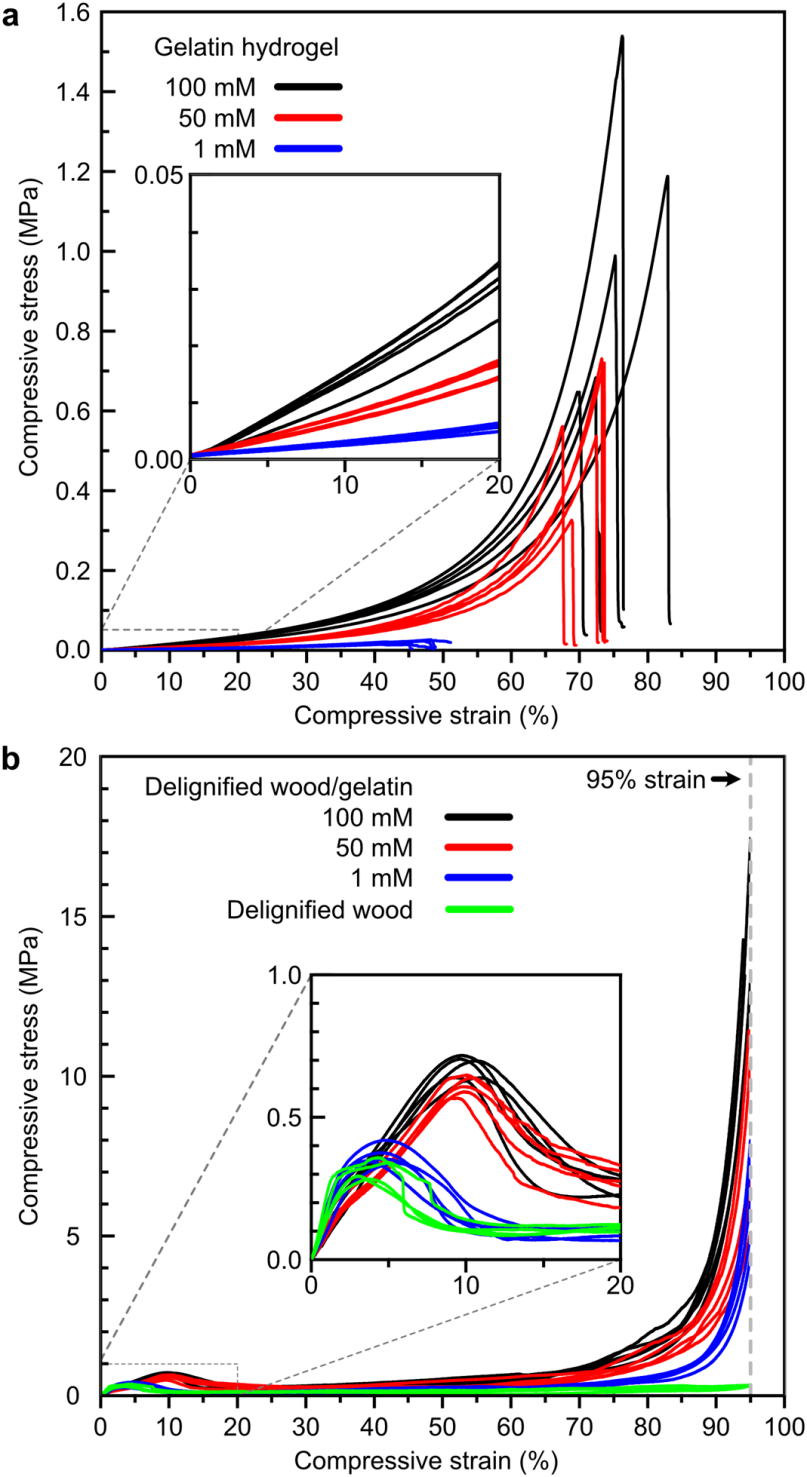
Compressive stress–strain curves of (**a**) genipin crosslinked gelatin hydrogels and (**b**) delignified wood and delignified wood/gelatin hydrogels at genipin concentrations of 1, 50, and 100 mM.

The typical compressive stress–strain curves of gelatin, delignified wood, and delignified wood/gelatin hydrogels are shown in Fig. 5 and their mechanical property data are summarized in Table 3. When neat gelatin was chemically crosslinked with genipin, the enhancement in elastic modulus, fracture stress and fracture strain was achieved owing to higher crosslinking density with increasing concentration of genipin (Table 3). The elastic modulus of gelatin hydrogel increased from 22.5 ± 2.4 kPa to 135.8 ± 23.0 kPa as the genipin concentration increased from 1 to 100 mM, respectively. The crosslinked gelatin hydrogel showed elastic behaviour and large deformation (compressive strain up to 50%) was induced with low stress load (Fig. 5a). Increasing crosslinking density resulted in denser polymer network with decreased water content (from 88.2 ± 0.1 to 85.9 ± 0.1 wt%, Table 1), which substantially enhanced the mechanical properties of neat gelatin hydrogel^27^. As the genipin concentration increased from 1 to 100 mM, significant increase in fracture strain and fracture stress for neat gelatin hydrogel was observed (Table 3). The impact of crosslinking density on the mechanical properties of gelatin is significant due to its amorphous nature and lack of rigidity^27^. Relatively high fracture stress (1.01 ± 0.37 MPa) was achieved at a fracture strain of 75.3 ± 4.9% by using 100 mM genipin.

**Table 3:** Effect of genipin concentration on the compressive mechanical properties of delignified wood, gelatin, and their composite hydrogels.

Sample	Genipin (mM)	Elastic modulus (MPa)	Fracture strain (%)	Fracture stress (MPa)	Yield stress (kPa)	Yield strain (%)
Gelatin hydrogels	1	0.0225 ± 0.0024	45.4 ± 3.6	0.0217 ± 0.0046	–	–
	50	0.0663 ± 0.0064	71.2 ± 2.8	0.5742 ± 0.1654	–	–
	100	0.1358 ± 0.0230	75.3 ± 4.9	1.01 ± 0.37	–	–
Delignified wood	–	21.89 ± 3.76	> 95	0.3146 ± 0.0072^a^	311.0 ± 21.6	3.2 ± 0.3
Delignified wood/gelatin hydrogels	1	18.12 ± 1.87	> 95	5.06 ± 1.05^a^	382.3 ± 32.8	4.5 ± 0.2
	50	14.07 ± 1.42	> 95	7.40 ± 2.29^a^	652.6 ± 49.8	9.7 ± 0.2
	100	11.82 ± 1.51	> 95	14.44 ± 2.65^a^	689.3 ± 34.9	10.2 ± 0.6

^a^Compressive stress at the compressive strain of 95%.

By contrast, the delignified wood hydrogel showed a completely different compressive stress–strain curve with an initial linear elastic deformation followed by a non-linear plastic yielding and the highest stress (311.0 ± 21.6 kPa) was reached at a compressive strain of 3.2 ± 0.3% (Fig. 5b). Due to the misalignment of fibres, shear force was generated along with compression, thus tangential displacement of fibres under stress transitioned into a plateau when the strain was between 5 and 90%. Benefit from the crystalline nature of cellulose and oriented organization of cellulose microfibrils along axial direction in the secondary cell wall of wood^40^, high stiffness was achieved with an elastic modulus of 21.89 ± 3.76 MPa for the delignified wood hydrogel. When gelatin was infiltrated into delignified wood and crosslinked by genipin, the composite hydrogels showed similar compressive stress–strain profile as delignified wood, and a decrease in elastic modulus with an enhancement in yield stress and an increase in yield strain were observed (Table 3). Although the composite hydrogels had much lower water content (higher solid density) as compared to delignified wood (Table1), the elastic modulus of the composite hydrogels decreased with the increasing genipin concentration as compared to delignified wood. This suggests that the volume fraction of infiltrated gelatin inside the wood cell walls and cell wall corners were increased with increasing crosslinking of gelatin. Indeed, the interaction between gelatin and delignified wood was also enhanced with increasing genipin concentration as confirmed by FTIR analysis. When 1 mM genipin was applied, slight increase in both yield stress and yield strain was found for composite hydrogel as compared to delignified wood. As the concentrations of genipin were increased to 50 and 100 mM, the yield stress of delignified wood/gelatin hydrogel increased two-fold compared to delignified wood to 652.6 ± 49.8 kPa and 689.3 ± 34.9 kPa, respectively, owing to the dense crosslinking between amino acid chains of gelatin and subsequent formation of hydrogel network within the cell lumens of delignified wood as revealed in Fig. 4e–h. The network structure inside wood cell lumens contributed to energy dissipation during compression, improving the resistance of composite hydrogel to yield, hence the yield strain was increased to 9.7 ± 0.2% and 10.2 ± 0.6%, respectively. The fracture strains of delignified wood and the composite hydrogels are all higher than 95%. The compressive stress of the composite hydrogels at 95% compressive strain was increased significantly with increasing genipin concentration as compared to that for delignified wood. With 100 mM genipin, the compressive stress at 95% compressive strain was as high as 14.44 ± 2.65 MPa, one to two magnitude higher than that for neat delignified wood and the fracture stress for neat gelatin crosslinked with 100 mM genipin (Table 3). The elastic modulus of the composite hydrogel (11.82 ± 1.51 MPa) was also two magnitude higher than that for neat gelatin hydrogel at 100 mM genipin. Such synergistic enhancement in both elastic modulus and compressive stress indicated the mutual toughening effect owing to the formation of porous structure of gelatin in the lumens of honeycomb like wood cell structure and strong intermolecular hydrogen bonding between infiltrated gelatin and delignified wood inside the wood cell walls and cell wall corners.

### Swelling behaviour

**Figure 6:**
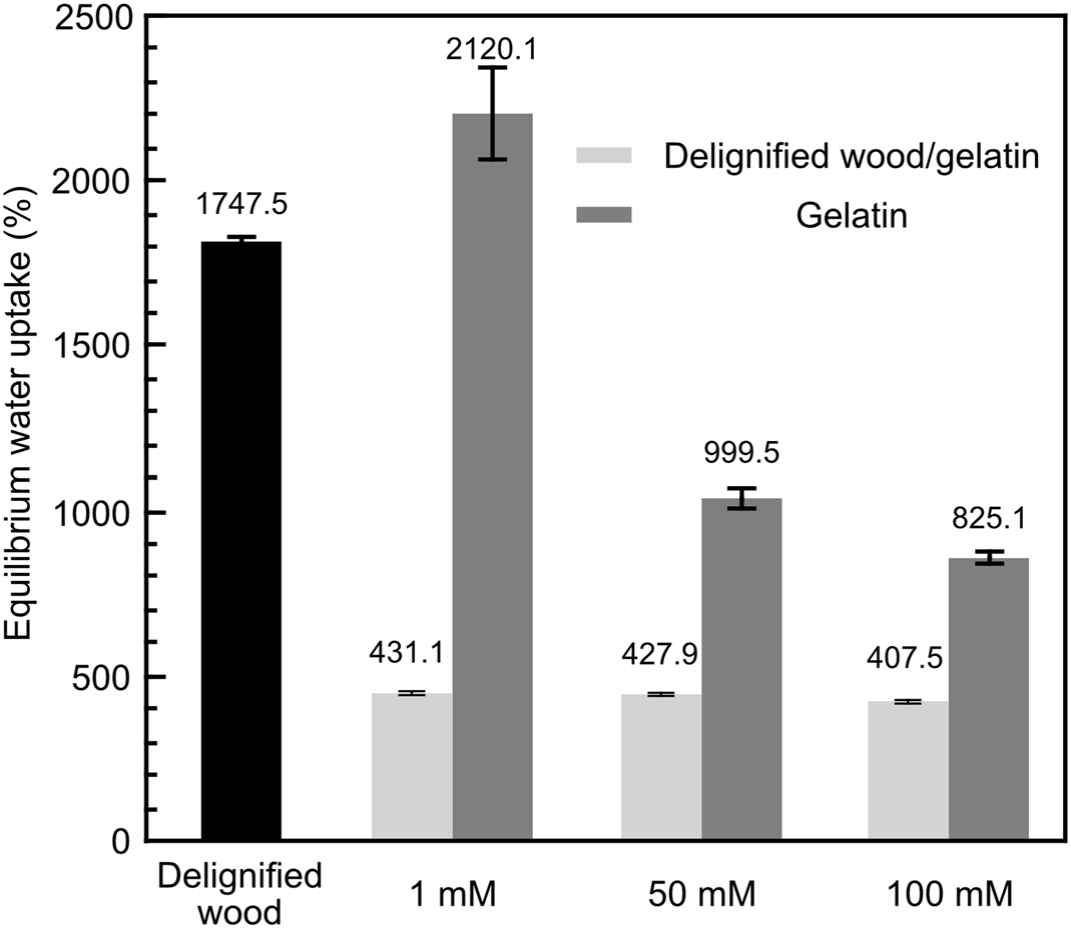
Effect of genipin concentration on the equilibrium swelling ratio of delignified wood, delignified wood/gelatin hydrogel and gelatin hydrogels in PBS buffer solution.

The swelling behaviour is another key factor which is strongly influenced by the structure of composite hydrogel. To study the swelling behaviour of neat gelatin hydrogel, delignified wood and composite hydrogels, samples were freeze-dried and then rehydrated in 0.1 M PBS (pH 7.4) buffer at 37 °C for 48 h to reach an equilibrium state. As shown in Fig. 6, the equilibrium water uptake of delignified wood was 1747.5 ± 14.1% because of its high porosity and hydrophilicity. Freeze-fried neat gelatin hydrogel also showed high water uptake, around 2120.1 ± 153.9%, when 1 mM genipin was applied. The loosely crosslinked gelatin network enabled accommodating large volume of phosphate buffer. The swelling of neat gelatin hydrogel was restricted when higher concentration of genipin was applied. A water uptake of 825.1 ± 17.7% was obtained at a genipin concentration of 100 mM. A plausible explanation is the formation of a rigid network through genipin crosslinking, which reduced the accessible hydrophilic groups as well as the extensibility of gelatin network. Different from neat gelatin, delignified wood/gelatin hydrogels showed less dependence on the genipin concentration. The water uptake values of the composite hydrogels were 431.1 ± 5.7%, 427.9 ± 4.4%, and 407.5 ± 5.2% at a genipin concentration of 1, 50, and 100 mM, respectively. Such lower water uptake values as compared to delignified wood and neat gelatin were resulted from the infiltration of gelatin into wood cell wall and the formation of strong intermolecular hydrogen bonding between delignified wood and gelatin after genipin crosslinking. These values also suggested that the corresponding water content of the PBS rehydrated composite hydrogels were ca. 81.2%, 81.1%, and 80.3%, respectively, same to the original water content in the composite hydrogels (Table 1). This indicates that unlike the neat gelatin hydrogel, further swelling of the composite hydrogel did not occur. Indeed, the accessibility of delignified wood cell wall and hydrophilicity of both components were significantly constrained. Particularly, the rigid nature of the cell wall structure in delignified wood restricted the swelling of gelatin.

As a comparison, when 30 wt% gelatin was crosslinked with *N*-(3-dimethy-laminopropyl)-*N*′-ethylcarbodiimide hydrochloride (EDC) within the bacterial cellulose (BC) scaffold^27^, the double-network (DN) composite hydrogel showed a compressive stress of 3.7 MPa, a modulus of 1.7 MPa, and an a degree of swelling of 5.8, which is equal to a water uptake value of 480%. Another example is TEMPO-oxidized CNF/gelatin (1:10 by weight) composite, in which gelatin was crosslinked initially through physical dehydrothermal treatment and then chemically with 6 wt% hexamethylenediamine and/or 1 wt% genipin. The resulted composite hydrogel showed a compressive stress of 25.6 kPa, an elastic modulus of 35.2 ± 3.1 kPa and a swelling ratio of 10.8 ± 0.6, which is equal to a water uptake value of around 1080%^8^. Photo-crosslinked IPN hydrogel consisting of chitosan and gelatin methacryloyl achieved a compressive modulus of 116.08 ± 9.62 kPa and an equilibrium water uptake value of around 1000%^19^. Taking advantage of the native aligned cellulose fibrils in wood cell wall and honeycomb like cellular structure of wood^5^, the delignified wood/gelatin hydrogel prepared in this work was proved to be both mechanically tough under compression (Fig. 5) and highly resistant to swelling under physiological condition (Fig. 6). Besides, the water content of delignified wood/gelatin hydrogel (ca.81%, Table 1) is among the highest which is close to that in natural cartilage^21^.

## Conclusions

A bio-based composite hydrogel composed of gelatin and delignified wood was successfully prepared through infiltration of gelatin into the porous wood template at 50 °C and subsequent crosslinking with genipin at 4 °C. The composite hydrogels demonstrated tunable porous structure with high mechanical strength and high resistance to swelling during rehydration in phosphate buffer (0.1 M, pH 7.4) at 37 °C. With increasing genipin concentration from 1 to 100 mM, the composite hydrogels showed an increase in compressive yield stress as high as 689.3 ± 34.9 kPa owing to the infiltration of gelatin into the cell wall and cell wall corner and the formation of gelatin network within lumen of the well-preserved wood cell structure in delignified wood. The elastic modulus of the composite hydrogel was 11.82 ± 1.51 MPa at a genipin concentration of 100 mM, two magnitude higher than the corresponding neat gelatin hydrogel. The swelling of gelatin was confined within lumen of the wood cell, resulting in the water uptake at equilibrium state as low as 407.5 ± 5.2%. These unique properties are superior to those for the DN hydrogel of BC/gelatin and IPN hydrogels of CNF/gelatin and chitosan/gelatin. In addition, both pore size of the infiltrated gelatin and porosity of the freeze-dried composite hydrogels were tunable by varying the genipin concentration. This work demonstrated wood structure an excellent template in preparation of high-performance composite hydrogels, taking advantages of inherently aligned CNFs and native porous cell wall structure for mechanical reinforcement and structural confinement.

## Methods

### Reagents and materials

Gelatin from bovine skin (75 Bloom), sodium chlorite, sodium acetate, acetic acid, glycine, sodium dihydrogen phosphate dihydrate and sodium phosphate dibasic dihydrate were purchased from Sigma-Aldrich and used as received. Genipin powder (98%, HPLC) was a product of Zhixin Biotechnology, China. Wood blocks (10 × 10 × 10 mm^3^, *Longitudinal* × *Radial* × *Tangential*) were cut from air-dried balsa wood (*Ochroma pyramidale*) sticks with a density of 175 kg/m^3^ purchased from Wentzel’s Co. Ltd., Sweden.

### Preparation of delignified wood

Balsa wood blocks were immersed in 1 wt% sodium chlorite buffered with acetate solution (PH 4.6) overnight at room temperature prior to heating up. The delignification was then carried out at 80 °C for 24 h. After washing thoroughly with deionized water, the delignified wood blocks were kept in water until further use.

### Preparation of delignified wood/gelatin composite hydrogels

Gelatin was dissolved in deionized water at concentration of 30 wt% and stored overnight at 50 °C. The delignified wood blocks were then transferred into the gelatin solution and kept for 1 week at 50 °C to ensure a complete infiltration of gelatin into the delignified wood. Gelatin-impregnated wood samples were then taken out and cooled down to 4 °C overnight to allow physical crosslinking of gelatin. The samples were then crosslinked in a genipin solution with different concentrations: 1, 50, and 100 mM at 4 °C for 48 h. The composite hydrogels were then washed with 1 wt% glycine solution followed by deionized water to remove the unreacted genipin. Neat gelatin hydrogel was prepared in the same way as the composite hydrogel.

### Attenuated total reflection Fourier transform infrared spectroscopy (ATR-FTIR)

ATR-FTIR spectra of original wood, delignified wood, delignified wood/gelatin hydrogel, and neat gelatin without genipin crosslinking were obtained with a FTIR spectrometer (Spectrum 2000, Perkin-Elmer, USA). The resolution was 4 cm^-1^ and 32 scans were accumulated over a range of 600–4000 cm^-1^ for each sample. Prior to FTIR characterisation, thin slices of each samples were obtained with microtome and freeze-dried.

### Optical microscopy and FE-SEM

The morphology of crosslinked gelatin within the delignified wood structure was characterized by using an optical microscope (LSM 510 Pa, Zeiss, Germany) in wet state. The microstructure was further characterized by using a field emission scanning electron microscope (FE-SEM, S-4800, Hitachi, Japan) working at low acceleration voltage (3 kV) and short working distance (8 mm). Thin Sections (200 μm in thickness) were obtained from the cross section of the delignified wood/gelatin hydrogel and delignified wood with a sliding microtome (SM 2010R, Leica, Germany). The hydrogel sections were then frozen at − 20 °C for 24 h and lyophilized with a freeze-dryer for 48 h. Freeze-dried sections were fixed onto metal stubs with carbon tape and then sputtered with platinum-palladium with a thickness of 3 nm. Pore size of the infiltrated gelatin in the freeze-dried composite hydrogel was measured from the FE-SEM images using Image-J software and 100 pores were measured for each sample^41,42^.

### Compression test

Compression test was carried out on a universal mechanical tester (Instron-5566, Instron, USA) equipped with a 10 kN loading cell. The strain rate was 1 mm/min. Both gelatin hydrogel and delignified wood/gelatin hydrogels were taken out from water and tested immediately after wiping off the excessive water on the surface. Load was applied to the composite hydrogel samples along the longitudinal direction of the wood. At least five specimens were tested for each sample.

### Swelling behaviour

Delignified wood, delignified wood/gelatin hydrogels and neat gelatin hydrogels were freeze-dried and then rehydrated in phosphate buffer (0.1 M, pH 7.4) at 37 °C for 48 h. The equilibrium water uptake (*Q*) was calculated using the following equation:

1 $${\text{Q }} = {\text{ }}\frac{{W_{e} - W_{d} }}{{W_{d} }} \times 100\%$$

where *W*_*e*_ is the weight of the sample at equilibrium condition and *W*_*d*_ is the weight of the dry sample.

### Porosity

The porosity of freeze-dried composite hydrogels was estimated based on their bulk density (*ρ*_*b*_) calculated from dry weight and apparent volume (geometry), and true density (*ρ*_*t*_) measured with a helium pycnometer (AccuPyc 1330, Micromeritics, Norcross, GA, USA), using the following equation:

2 $${\text{porosity}} = \left( {1 - \frac{{\rho _{b} }}{{\rho _{t} }}} \right) \times 100\%$$
